# Modification of Hydrogenation and Corrosion Properties of Hydrogen Storage Material by Amorphous TiCrFeCoNi HEA Layer

**DOI:** 10.3390/ma15072593

**Published:** 2022-04-01

**Authors:** Agnieszka Giemza, Maria Sozańska, Henryk Bala

**Affiliations:** 1 Faculty of Production Engineering and Materials Technology, Częstochowa University of Technology, Al. Armii Krajowej 19, 42-200 Częstochowa, Poland; agnieszka.giemza@pcz.pl; 2 Faculty of Materials Engineering, Silesian University of Technology, Krasińskiego 8, 40-019 Katowice, Poland; 3 Faculty of Science and Technology, Jan Długosz University in Czestochowa, Al. Armii Krajowej 13/15, 42-200 Częstochowa, Poland; h.bala@ujd.edu.pl

**Keywords:** hydrogen sorption/desorption, high entropy alloy, HEA, surface modification

## Abstract

The effect of encapsulation of LaNi_4.5_Co_0.5_ powdered hydrogen storage material with ≈0.5 µm thick, magnetron-sputtered amorphous film of TiCrFeCoNi high-entropy alloy (HEA) on functional hydrogenation parameters of the hydride electrode is discussed. The multicycle galvanostatic charge/discharge tests carried out in deaerated, 6 M KOH solution allow for determining specific capacity decrease, exchange current density of the H_2_O/H_2_ system, and high rate discharge ability (HRD) of the hydride electrodes. Concentrations of individual constituents of the HEA in the particle coating determined by EDS analysis were practically the same (≈20 at.%) as in the applied TiCrFeCoNi target material. The XRD phase analysis pointed out the amorphous structure of the HEA coating. The presence of HEA coating decreases capacity by 10–15 per cent, but increases exchange current density for H_2_O/H_2_ system. The effect of HEA on capacity fade is ambiguous: low for 10–25 cycles (most probably due to effective corrosion inhibition) and distinct at long-term cycling (most probably due to galvanic effects resulting from mechanical degradation of particle surface). The presence of HEA coating considerably improves the HRD of the electrode material: for a discharge rate of 5C, the HRD coefficient becomes 4.6 times greater for HEA modified storage material.

## 1. Introduction

High-entropy alloys (HEAs) [1,2,3,4,5,6], with equiatomic or near-equiatomic multicomponent structures, have become widely used in the design and construction of elements that require such mechanical and chemical material characteristics as high strength, resistance to abrasion, good thermal (oxidation) stability, catalytic properties, or corrosion resistance. HEAs found numerous applications in manufacturing a variety of functional industrial devices such as moulds, dies, machinery parts, pipes, pump elements, etc. The concept of multiprincipal element alloys (MPEAs) and high-entropy alloys (HEAs) was realised over 15 years ago, first reported in 2004 by scientific teams of Cantor [1] and Yeh [2]. Yeh et al. [2,7,8] indicate that the unique properties of HEAs are a result of four main factors: high entropy, slow diffusion, lattice distortion, and the so-called cocktail effect (even “mixing” of the properties). Due to their high mixing entropy, HEAs tend to form simple solid solution phases of *fcc*, *bcc*, or *hcp* rather than the complex or intermetallic phases [5]. The unique properties of HEAs have made them a promising material for use also in the form of thin coatings of different functional materials, which additionally expands the prospects of their extensive use [4,5,7,8,9,10]. High entropy coatings are often composed of such transition metals as Ni, Cr, Co, Ti, or Cu (metals with relatively positive standard electrode potentials or spontaneously passivating in aqueous media). Therefore HEAs (often consisting also of Al addition), based on the above-mentioned metals, possess not only excellent mechanical properties but also show good corrosion resistance (including high temperature and chemically aggressive environments) [2,3,4]. Chen et al. [11] applied 6 and 7 component targets with formal stoichiometry of FeCoNiCrCuAlMn and FeCoNiCrCuAl_0.5_ and magnetron sputtering technique to manufacture corresponding surface layers. The phase structure of obtained layers showed a chemical composition similar to that of the goal, and the layer structure was close to nanocrystalline. However, as the concentration of flowing nitrogen increased, the authors observed a distinct tendency of the HEA coating to amorphisation. Huang et al. [12] studied the structure, hardness, and phase stability of the AlCoCrCu_0.5_NiFe coating. In the absence of oxygen in the working gas stream, the coating was amorphous. The addition of >10% of oxygen to the working gas was prone to crystallisation of the deposited coating [12].

The deposition of intermetallic alloy films (including HEAs) has been reported using various physical vapour deposition (PVD) techniques such as pulsed laser deposition, laser cladding, ion sputtering, ion plating, RF-magnetron sputtering, or flash evaporation-deposition [6,13,14,15,16,17,18,19]. Amongst multicomponent thin films on different alloy substrates and powders are also thin films of hydrogen storage materials (HSMs, e.g., LaNi_5_ type) on different substrates [13,19,20,21,22]. The utilisation of sputtered intermetallic thin films of HSMs is considered beneficial because of their higher thermal conductivity, improved resistance to hydrogen pulverisation, and high efficiency of protective and catalytic layers as compared with their bulk counterparts [13,19,23,24]. The crystallinity and capacity of the PVD thin films depend on the type of goal, kind of substrate, and sputter parameters. The sputtered films of HSMs are usually amorphous and generally show about two times lower capacities than the respective bulk crystalline alloys. Some of the authors think that this is due to the restricted volume expansion within the film since a peeled-off film could absorb almost the same amount of hydrogen [13,20,23]

Magnetron sputtering of thin, Ni-based layers on different HSM particles is more and more widely applied in the latest published reports [18,25,26,27,28]. Ni, similarly to Co and Fe, has well-documented catalytic properties toward H_2_O/H_2_ redox system; therefore, the nickel-modified surface of HSMs improves material activation and increases the exchange current density of the hydrogen electrode [27,28]. Additionally, Ni belongs to relatively noble elements; therefore, tight and compact nickel coatings (cathodic films) should effectively protect more active substrates against electrochemical corrosion. One should, though, remember that the presence of discontinuities in cathodic films may release galvanic effects and accelerate local corrosion phenomena within the substrate [29]. For example, galvanic (bimetallic) corrosion causes greater capacity fade and cycle life shortening for Ni-encapsulated, powdered (La,Mg)_2_Ni_7_-based HSMs [27,28].

In this article, the subject of research was the modification of the hydrogenation and corrosion properties of the hydrogen storage material by the amorphous alloy layer with high entropy TiCrFeCoNi. In particular, the surficial modification of powdered (20–50 µm particles) hydrogen storage material with LaNi_4.5_Co_0.5_ stoichiometry (based material) has been accomplished. As a target material, the TiCrFeCoNi high-entropy alloy (equimolar, sintered material) has been used. As it has been mentioned, the multicomponent metallic coating should facilitate the maintaining of high specific capacities of the hydrogen storage substrate and reveal electrocatalytic properties to hydrogen sorption, and it should protect the active material particles (containing highly active lanthanum) against corrosion attack of aqueous, alkaline electrolyte. Among the constituents of the arranged HEA composition, Ni, Co, and Fe are responsible for catalytic behaviour [18,25,26,27,28,30], whereas Cr and Ti, due to their tendency to effective passivation of HSM [6,31], for corrosion resistance, and thus prolonged cycle life. The characteristics of changes in hydrogenation properties of hydrogen storage material by the amorphous layer of high-entropy TiCrFeCoNi alloy were accompanied by the morphology of the tested LaNi_4.5_Co_0.5_ powders before and after the HEA modification process (SEM), the chemical composition (EDS), and phases analysis (XRD) of amorphous structure of the HEA coating.

## 2. Materials and Methods

The commercially available (American Elements, Los Angeles, CA, USA), *CaCu*_5_ type compound with LaNi_4.5_Co_0.5_ composition has been chosen as the active HSM of the substrate. The XRD examinations of the based material have been presented in [32]. Small pieces (3–6 mm) of LaNi_4.5_Co_0.5_ alloy were mechanically crushed, then milled (Fritsch, Pulverizette mill, Ar atmosphere) and sieved to separate the powder fraction of 20–50 µm. The TiCrFeCoNi thin films were deposited on the powder particles using a 30 mm diameter HEA target in the Dora Power Pack system [33]. The high-entropy alloy target applied in this study was manufactured by powder metallurgy route: thoroughly mixed metallic powders with intentional molar proportions of elements (Ti, Cr, Fe, Co, and Ni, each of 20.0 at.%) were mechanically pressed and sintered. The target test portion has been dissolved in HCl solution and analysed using the inductively coupled plasma (ICP) spectrometry technique. Contents of the individual elements in the synthesised target were equal to their intentional amounts with a relative accuracy better than 0.1%. The amounts of 5 g of tested powder were inserted into the drum-shaped rotary stage, rotating with a rate of 26 rpm, and then magnetron sputtered right through 2 h. During sputtering, the substrate temperature was about 50 °C, whereas the dynamic vacuum (pressure taken from Ar-gas) in the reaction chamber was on the level of 0.5 ± 0.2 Pa.

The electrode pellets (*Φ* = 5 mm, *h* ≈ 0.4 mm, the mass of active material ≈30 mg, determined with an accuracy of 0.1 mg for each individual pellet) have been prepared by careful mixing of the HEA-sputtered powder with 5 mass % of C-graphite (conducting powder), 10 mass % of poly(vinylidene fluoride) (PVDF) as a particle binder, and a small amount of acetone to ensure thick paste consistency. After acetone evaporation, the homogenised mass was pressed with 50 bar force, analogously to previously published papers [34,35,36,37,38].

The morphology and chemical composition of the magnetron-sputtered HEA layers on HSM material were investigated using Hitachi S-3400N scanning electron microscope (SEM) coupled with Thermo Noran X-ray energy dispersion spectrometer (EDS) and the Seven X-ray microanalysis system. The HEA-sputtered powder sample particles were located for testing on graphite pads and isolated with the carbon-rich epoxy resin composition. After polishing, the distribution of chemical elements was given using the X-ray microanalysis method with an EDS spectrometer on the cross-sections of samples in selected microareas.

In order to determine the phase composition of the obtained materials, diffractometric studies were carried out using a Bruker D8 Advance diffractometer equipped with a Johansson monochromator (λCu Kα1 = 1.5406 Å) and LYNXEYE strip detector. The obtained diffractograms were analysed using the PDF4 + database.

The pellets have been used as working electrodes (pellet sides isolated with epoxy resin, pellet back covered with silver glue, acted as a current collector). The electrode’s porosity, in terms of electrolyte accessibility to HSA material particles within the pellet, was approximately 7 vol.% [39]. The charge/discharge tests have been carried out in a 50 mL Teflon cell equipped with Au-auxillary- and HgO/Hg reference electrodes using Ar-saturated 6 M KOH solution at a temperature of 22 ± 0.2 °C. The CHI Instruments electrochemical workstation (Austin, Texas) has been applied for galvanostatic and potentiostatic experiments. In charge/discharge cycling, the electrodes were cathodically charged with the current density of −186 mA∙g^−1^ (−0.5*C* rate) during 9000 s for each cycle. The discharge process continued with a rate of +186 mA∙g^−1^ (+0.5*C* rate) until electrode potential reached *E* = −0.50 V vs. HgO/Hg (cutoff potential), analogously as described in [34,35,36,37,38]. The electrode charging/discharging has been carried out up to *N* = 60 cycles (for cycle numbers greater than 65–70, the worsening of electrode coherence occurred which was accompanied by partial loss of particles compactness with binder).

## 3. Results

### 3.1. Evaluation of the Morphology and Chemical Composition of HEA-Modified LaNi_4.5_Co_0.5_ Powder Particles

**Figure 1:**
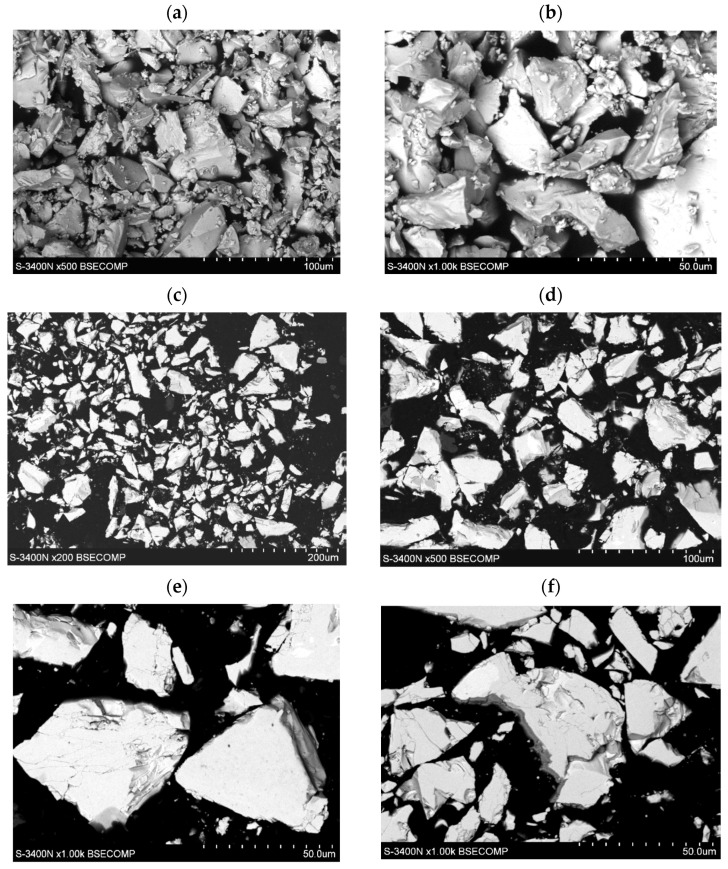
Evolution of morphology of the LaNi_4.5_Co_0.5_ particles: (**a**,**b**) before modification, (**c**–**f**) after coating with a TiCrFeCoNi layer of the applied HEA.

The morphology of the tested LaNi_4.5_Co_0.5_ powders before and after the HEA modification process is shown in Figure 1. The powders had very irregular shapes in the form of polyhedrons of very different sizes of walls, the largest diameters of the walls varied from several hundred nm to 50 µm (Figure 1a,c). The surface of the powder shows microcracks and faults in many places (Figure 1e,f). In the images of LaNi_4.5_Co_0.5_ particles (metallographic section) after the HEA modification process, a clear differentiation in grey levels can be seen. This is an effect resulting from the difference in the average chemical composition between adjacent microareas (Figure 1d–f). Darker areas have a lower average mass chemical composition. The result is a directly applied technique of obtaining an image in a scanning electron microscope (SEM)—it is an image from the BSE COMP detector *(Back Scattered Electron detector in Composition*).

**Figure 2:**
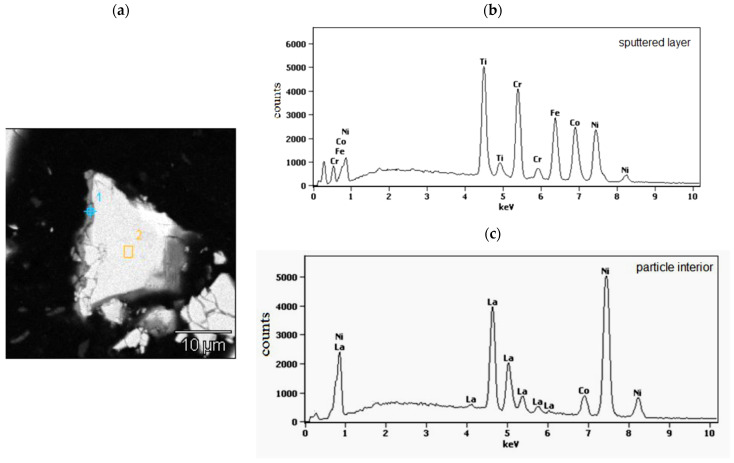
(**a**) Polished cross-section of isolated LaNi_4.5_Co_0.5_ powder particle coated with the magnetron sputtered TiCrFeCoNi layer. The numbers denote EDS analysis areas: 1-HEA layer and 2-particle interior; (**b**,**c**)-the EDS spectra from the particle surface layer (point 1 in (**a**)) and particle interior (area 2 in (**a**)).

In Figure 2, as the example, the cross section of isolated, representative powder particle of the LaNi_4.5_Co_0.5_ material coated with a TiCrFeCoNi layer of the applied HEA is presented. On the left side of the particle, a grey layer, about (0.4–0.8) μm thick, is visible. The places of EDS analysis are labelled with numbers 1 (sputtered layer) and 2 (particle interior). The corresponding EDS spectra are shown in Figure 2b,c and the average chemical compositions for these areas are summarised in Table 1.

As it can be seen from SEM/EDS data (Figure 2b,c, Table 1), the sputtered layer (point 1) contains all elements of the goal material (Ti, Cr, Fe, Co, and Ni) in practically equimolar proportions: (19.9 ± 3.8) at.%. This confirms the lack of preferences in the deposition of individual components during the magnetron sputtering process. On the other hand, analysis of the interior of the alloy displays only the presence of metals belonging to the tested hydrogen storage alloy. The molar proportions of these elements in area 2 are close to those resulting from the alloy stoichiometry.

### 3.2. Phase Analysis of the Tested Powder Samples

**Figure 3:**
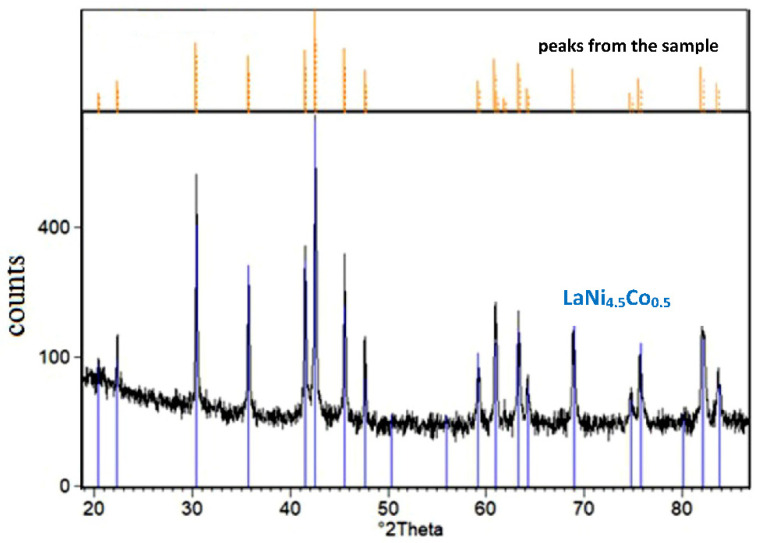
XRD spectrum of the tested LaNi_4.5_Co_0.5_ powder sample modified by magnetron sputtering of TiCrFeCoNi HEA layer (comparison of the X-ray spectrum for the sample before and after the HEA process).

Figure 3 shows the XRD spectrum of LaNi_4.5_Co_0.5_ powder material covered with the TiCrFeCoNi coating.

For the tested LaNi_4.5_Co_0.5_ material modified by HEA sputtered layer, only the crystalline AB_5_ phase with a hexagonal structure was identified (*a* = 5.017 Å, *c* = 3.981 Å, space group: *P6/mmm* (191) [40]). No peaks from the coating material were revealed. As noted earlier, the deposited HEA layer was ≈0.5 µm thick, so in the case of the crystalline structure of the deposited layer, the presence of goal constituents should be revealed in the XRD pattern. Therefore, the absence of peaks coming from HEA constituents in Figure 3 indicates the amorphous character of the surface layer. It should be stressed that low-temperature magnetron sputtering of thin (up to 1 µm thick) metal layers on powder particles generally pursues amorphous layers on various substrates (intermetallic materials, compact glass, etc.). For example, amorphous layers of high purity nickel were obtained by our team for a series of Mg-rich La_2_Ni_7_-based hydrogen storage alloys synthesised by mechanical alloying [25,27,28].

### 3.3. Effect of HEA Sputtering on Discharge Capacity of the Tested HSM Electrodes

**Figure 4:**
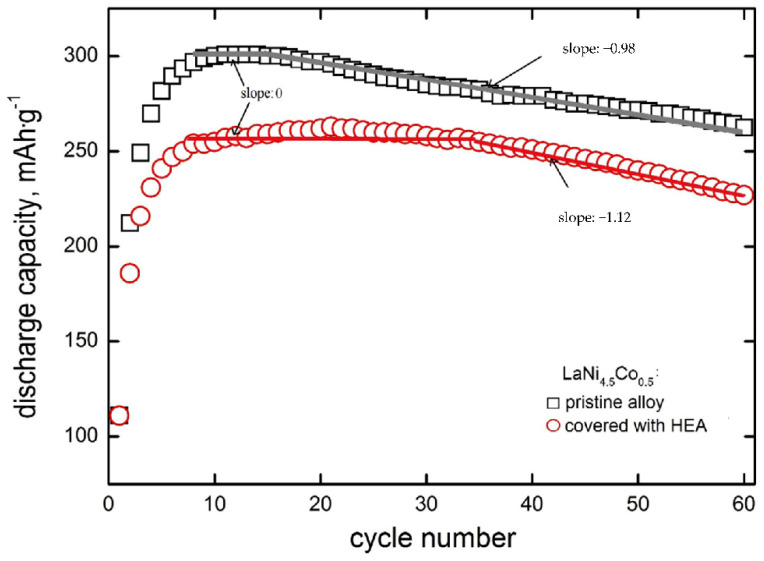
Discharge capacities as a function of cycle number for electrodes made of based LaNi_4.5_Co_0.5_ powder material and material modified with HEA.

In Figure 4, discharge capacities as a function of cycle number are presented for the tested powder composite electrodes made of (i) based LaNi_4.5_Co_0.5_ powder particles and (ii) particles modified by HEA sputtering.

It is seen that for both types of electrodes that the discharge capacity increases rapidly for the first 4–6 cycles. After *N* = 8–15 cycles, full activation of the based electrode material takes place, and then nearly linear capacity fade is visible in *Q*_disch_ = f(*N*) coordinate system with a slope of −0.98 mAh∙g^−1^∙cycle^−1^. For HEA modified material, capacities are generally lower by 15–20%, and the range of practically constant capacity is distinctly wider (it occurs between *N* = 8–35 cycles) in case of lack of capacity fade, i.e., when electrode corrosion rate (*r*_corr_) is close to zero [37,38]. This means that HSM particles modification by HEA sputtering strongly inhibits corrosion degradation up to 35 cycles. Rectilinearity of *Q*_disch_ = f(*N*) dependence testifies to zero-order kinetics of the corrosion process [41,42]. The absolute slope of this dependence (*y* = *a* − *bx* type) is equal to *b* = *k*_M_*k*_0_ (where *k*_M_ equals 60.0 mA∙h∙cm^3^∙g^−2^ [41] and *k*_0_ is zero-order rate constant). For zero-order kinetics, the reaction rate is constant within the whole rectilinear cycling range and equal to the rate constant (*r*_corr, 0_ = *k*_0_). Contrary to zero-order behaviour, capacity fade may also obey rectilinearity in the log*Q*_disch_ − *N* coordinate system (first-order kinetics) [37,38]. For the first-order kinetics, corrosion rate depends on the actual concentration of the HSM alloy undergoing corrosion (*r*_corr,I_ = *k*_I_∙*c*_M_), so the *r*_corr,I_ decreases with cycling. In practice, we exactly know only the initial concentration of the material in the electrode (*c*_M,init_). Therefore, for the first order processes, we determine initial corrosion rates from the capacity fade dependence: log*Q*_disch_ = log*Q*_disch,init_ − 0.434*k*_I_*N*. The material mass concentration, *c*_M_ depends on the electrode pellet preparation method. In case of method assumed in this work, initial material concentration is *c*_M,init_ = 6.2 g∙cm^−3^ (active powder mass per pellet volume) [37,38,41]. Corrosion rates of porous, powder composite electrodes of HSMs are expressed in g∙cm^−3^∙cycle^−1^, irrespectively for zero- or first-order kinetics processes. Starting with 35 cycles, an evident decrease in capacity appears for the HEA modified material (with a slope of −1.12 mAh∙g^−1^∙cycle^−1^). This means that after 35 cycles, corrosion of modified material occurs about 14% faster than that of based material. Formally speaking, the based material also exhibits a capacity plateau (and, thus, *r*_corr_ = 0), but it takes place in a much narrower cycling range (8 to 15 cycles—compare Figure 4).

In the case of based material, the linearity of the capacity decrease is observed starting from *N* = 15 cycles and the linear extrapolation (with a slope of −0.98 mAh∙g^−1^) to *N* = 0 determines the theoretical capacity of the unmodified material: *Q*_0_ = 318 mAh∙g^−1^. For electrodes with HEA coating, the slope of a rectilinear segment is approx. 15% greater than for the pristine material. Therefore, the TiCrFeCoNi coating does not exhibit protective properties in relation to the substrate. This is probably due to the weak compactness and, thus, noticeable porosity (permeability by electrolyte) of the HEA layer. Discontinuity of the HEA layer promotes undesirable galvanic effects: a spongy cathodic (more noble) coating locally accelerates the oxidation processes of the electrochemically more active substrate material. A similar phenomenon has been observed for Ni-coated nanocrystalline HSMs, containing highly active Mg addition of (La,Mg)_2_Ni_7_ type [27,28].

### 3.4. Effect of HEA Modification on Exchange Current Density of H_2_O/H_2_ System

**Figure 5:**
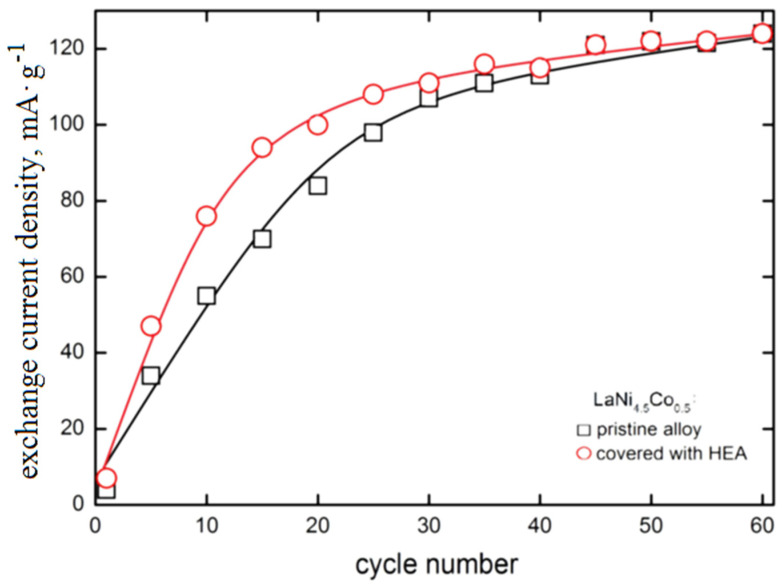
Exchange current density of H_2_O/H_2_ system as a function of cycle number for electrodes made of based LaNi_4.5_Co_0.5_ HSM material and HSM modified with HEA coating (6 M KOH, Ar, 22 °C).

In Figure 5, the exchange current density of the H_2_O/H_2_ system for HEA-modified HSM material versus electrode cycling is presented and compared with those of pristine alloy. The exchange current densities ($i_{H_{2}{O/H}_{2}}^{0}$) have been determined from potential-jump (∆*E*^j^) when the external current is switched from cathodic to the anodic direction at −0.5*C*/+0.5*C* charge/discharge rates, analogously as in Refs [37,38,43]. For the tested materials, the exchange current density increases with cycle number with an apparent tendency to settle on a constant level (≈120 mA∙g^−1^). The shape of measured dependences is similar to those we observed many times for different types of hydride electrodes [25,26,27,28,34,35,36].

**Figure 6:**
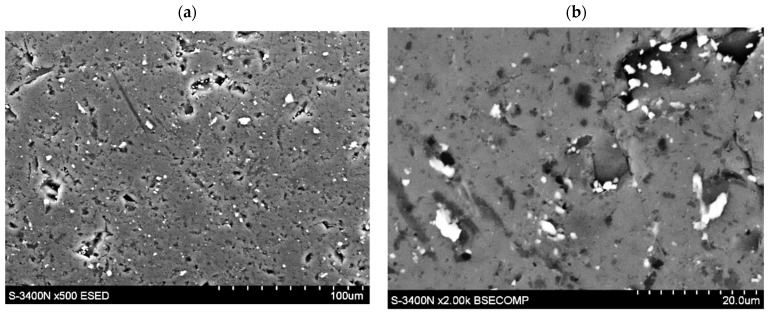
(**a**,**b**)-Local presence of discontinuities in TiCrFeCoNi layer of the applied HEA.

The presence of HEA coating particularly strongly increases the exchange currents for the first 30 cycles (for example, between the 10th and 20th cycle, the $i_{H_{2}{O/H}_{2}}^{0}$ is 35–40% greater for HEA modified material). However, for *N* > 30 cycles, the differences between values for based and HEA modified material gradually disappear. After *N* = 60 cycles both types of electrode materials reach values close to 120 mA∙g^−1^ (these values are relatively high, compared with other HSMs [30,32,34,39,42]). As seen, the advantageous effect of the increase due to HEA sputtering is observed for relatively short cycling only. For longer cycling exposure, galvanic corrosion effects evidently reduce the catalytic action of HEA film. Longer cycling reveals galvanic effects and local corrosion attacks, most probably due to the presence of discontinuities in HEA coating that facilitate permeability of the electrolyte through the surface layers (Figure 6).

**Figure 7:**
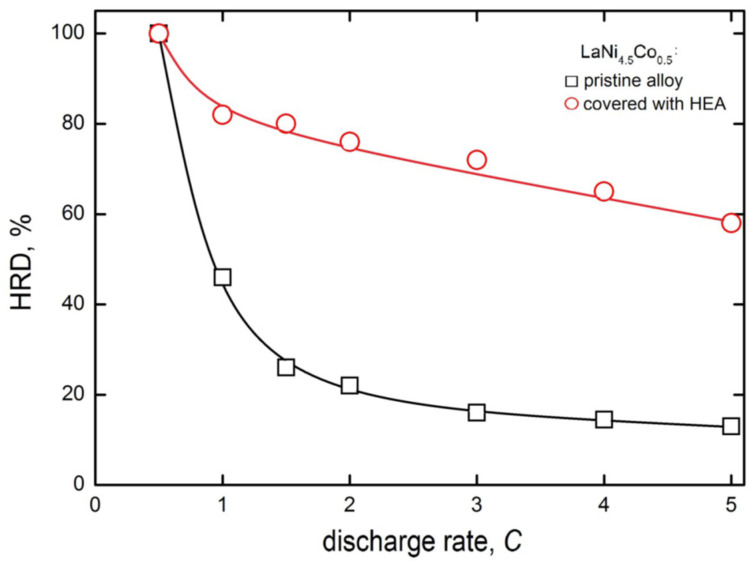
High rate dischargeability for electrodes made of pristine and HEA-modified LaNi_4.5_Co_0.5_ powders of HSM after 60 charge/discharge cycles (−0.5*C*/+0.5*C*) in 6 M KOH (Ar, 22 °C).

Figure 7 shows the high rate of dischargeability (HRD) behaviour of the electrodes made of pristine and HEA-modified powder HSM materials. The *HRD* parameter is defined here similarly as in [44,45], as the quotient of maximum discharge capacities at a given discharge rate (*i*_disch_) and at chosen, regular discharge rate (for example, 0.5*C*).

(1) $$HRD[\%]=\frac{Q_{i,\mathrm{disch}}^{\max}}{Q_{0.5C}^{\max}}\times 100$$

A consequence of such a definition is *HRD* = 100% for *i*_disch_ = 0.5*C*, and dischargeabilities are the lower, the greater the discharge rates. For greater discharge rates, due to lattice expansion (H-absorption) and rapid contraction (H-desorption), the stronger *HRD* decrease in greater discharge rates is attributed to faster mechanical degradation of HSMs at larger discharge rates [46]. Obviously, the greater the *HRD* parameter (for a given *i*_disch_), the more advantageous the hydrogen storage behaviour of the examined anode material.

It should be mentioned that in more practical works dealing with batteries, the high rate of dischargeability at a given discharge rate (*i*) is often defined in another way and symbolised *HRD*_i_ [46]. In the denominator of Equation (1), instead of residual capacity, e.g., *Q*_0.5C_, there appears a sum (*Q*_0.5C_ + *Q*_i_). Therefore, *HRD* > *HRD*_i._

Differences in *HRD* values for electrodes prepared from unmodified and HEA-sputtered powders become distinctly visible already at the 1*C* discharge rate, and the HRD drop is much more rapid for unmodified material. At the 5 C rate, the *HRD*_5C_ is 13% for based but nearly 60% for HEA-modified electrode material. This way, the presence of HEA coating on powder particles significantly limits the decrease in capacity at high discharge rates. This very advantageous performance is most probably the result of buffering action of crystal lattice expansion/contracting effects of the HEA coating

## 4. Conclusions

In this study, we analysed the modification of hydrogenation and corrosion properties of hydrogen storage material by its coverage with an amorphous TiCrFeCoNi high-entropy alloy layer. The general conclusions can be drawn as follows:

- The magnetron sputtering of high-entropy alloy with equimolar TiCrFeCoNi stoichiometry on active particles of hydrogen storage powder (LaNi_4.5_Co_0.5_) allowed to modify the particle surface with about 0.5 µm thick amorphous layers of the applied HEA;
- The analysis of the LaNi_4.5_Co_0.5_ particle morphology material coated with a TiCrFeCoNi layer of the applied HEA showed that the powders had irregular shapes, with numerous microcracks and faults, and their average diameters ranged from several hundred nm to 50 µm. The chemical composition of the coating was practically the same as the composition of the HEA target. The XRD phase analysis showed the amorphous structure of the HEA coating;
- The HEA-modified LaNi_4.5_Co_0.5_ material displays no capacity fade during the first 35 charge/discharge cycles in 6 M KOH. Longer cycling reveals galvanic effects and local corrosion attacks, most probably due to the presence of discontinuities in HEA coating that facilitate permeability of the electrolyte through the surface layers. As a result, the corrosion process of HEA-modified electrodes for longer cycling proceeds faster (by about 15%) than that of pristine material;
- HEA modification increases the exchange current density of the H_2_O/H_2_ system on the tested HSM electrode. This advantageous effect occurs up to 35 charge/discharge cycles and then disappears;
- HEA sputtering of LaNi_4.5_Co_0.5_ material particles significantly improves the high-rate dischargeability of the hydride electrodes: at a discharge rate equal to 5*C*, the HRD parameter is 4.6 times greater for the HEA modified material.

## Figures and Tables

**Table 1:** The average chemical composition (at.%) of the sputtered layer and particle interior determined by the EDS analysis (points 1 and area 2 in Figure 2a).

Element (wt.%)	La	Ni	Co	Fe	Cr	Ti
**Deposited layer (point 1)**	−	26.2	20.1	18.6	18.0	16.5
**Particle interior (area 2)**	13.1	78.2	8.2	−	−	−

## Data Availability

Not applicable.
